# Salsolinol Protects SH-SY5Y Cells Against MPP^+^ Damage and Increases Enteric S100-Immunoreactivity in Wistar Rats

**DOI:** 10.1007/s11064-022-03835-2

**Published:** 2022-11-30

**Authors:** Magdalena Kurnik-Łucka, Gniewomir Latacz, Joanna Goryl, Veronika Aleksandrovych, Krzysztof Gil

**Affiliations:** 1 Department of Pathophysiology, Faculty of Medicine, Jagiellonian University Medical College, Czysta 18, 31-121 Krakow, Poland; 2 Department of Technology and Biotechnology of Drugs, Faculty of Pharmacy, Jagiellonian University Medical College, Medyczna 9, Krakow, Poland

**Keywords:** Salsolinol, MPP^+^, SH-SY5Y cells, Wistar rats, S100, Enteric nervous system

## Abstract

A dopamine derivative, 1-methyl-6,7-dihydroxy-1,2,3,4-tetrahydroisoquinoline, known as salsolinol (SAL), has increasingly gained attention since its first detection in the urine of Parkinson’s disease patients treated with levodopa, and has been proposed as a possible neurotoxic contributor to the disease. Yet, so far, the neurobiological role of SAL remains unclear. Thus, the main aims of our study were to compare the neurotoxic potential of SAL with MPP^+^ (1-methyl-4-phenylpyridinium ion) in vitro, and to examine intestinal and metabolic alterations following intraperitoneal SAL administration in vivo. In vitro, SH-SY5Y neuroblastoma cell line was monitored following MPP^+^ and SAL treatment. In vivo, Wistar rats were subjected to SAL administration by either osmotic intraperitoneal mini-pumps or a single intraperitoneal injection, and after two weeks, biochemical and morphological parameters were assessed. SH-SY5Y cells treated with MPP^+^ (1000 μM) and SAL (50 µM) showed increase in cell viability and fluorescence intensity in comparison with the cells treated with MPP^+^ alone. In vivo, we predominantly observed decreased collagen content in the submucosal layer, decreased neuronal density with comparable ganglionic area in the jejunal myenteric plexus, and increased glial S100 expression in both enteric plexuses, yet with no obvious signs of inflammation. Besides, glucose and triglycerides levels were lower after single SAL-treatment (200 mg/kg), and low- to high-density lipoprotein (LDL/HDL) ratio and aspartate to alanine aminotransferases (AST/ALT) ratio levels were higher after continuous SAL-treatment (200 mg/kg in total over 2 weeks). Low doses of SAL were non-toxic and exhibited pronounced neuroprotective properties against MPP^+^ in SH-SY5Y cell line, which supports the use of SAL as a reference compound for in vitro studies. In vivo results give insight into our understanding of gastrointestinal remodeling following intraperitoneal SAL administration, and might represent morphological correlates of a microglial-related enteric neurodegeneration and dopaminergic dysregulation.

## Introduction

Salsolinol (1-methyl-6,7-dihydroxy-1,2,3,4-tetrahydroisoquinoline, SAL), since its first detection in the urine of Parkinson’s disease (PD) patients treated with L-DOPA (levodopa, L-3,4-dihydroxyphenylalanine), has been proposed as a possible neurotoxic contributor to the disease [1]. Further, the concept was strengthened by the discovery of the ability of SAL to form 1,2-dimethyl-6,7-dihydroxyisoquinolinium ions (DMDHIQ^+)^ considered to be analogous, due to basic nitrogen center, to 1-methyl-4-phenylpyridinium ions known as MPP^+^ [2–6]. Endogenously, SAL can be formed via non-enzymatic condensation of dopamine (DA) with acetaldehyde or pyruvate, and/or enzymatic synthesis from DA via (R)–SAL synthase [5]. Although MPTP (1-methyl-4-phenyl-1,2,3,6-tetrahydropyridine) and its metabolite MPP^+^ are well studied and recognized dopaminergic neurotoxins, neurodegenerative properties of exogenous SAL have also been numerously emphasized in vitro and in vivo [reviewed in 7]. So far, the majority of studies related to SAL have been brain-centered, yet the peripheral, and especially gastrointestinal (GI) consequences of SAL administration are worth further consideration [8] with regard to the gut-brain connection in the pathogenesis of PD [9]. So far, we have reported morphological alterations in myenteric neurons in LMMP (longitudinal muscle myenteric plexus) preparations and increased neuronal expression of the pro-apoptotic Bax protein after continuous intraperitoneal SAL administration (200 mg/kg in total over 2 or 4 weeks). We also observed an increased percentage of mean residual solid food in the stomach and decreased large intestine transit and water content of fecal matter as well as a decrease in body weight gain and slower adipose tissue accumulation after continuous intraperitoneal SAL administration, which may result from abnormal GI motility and absorption [10]. Those findings remain in agreement with Banach et al., who observed an impairment in the myoelectrical activity of intramuscular interstitial cells of Cajal in the GI tract of rats after intraperitoneal SAL (50 mg/kg/day over 3 weeks) injections [11]. What is more, SAL-treatment was also associated with increased serum levels of histamine and IL-1β [12]. Interestingly, SAL and its metabolites might possess both neurotoxic and neuroprotective properties. Initially, it was also reported that exogenous *(R)*-SAL (40 μM) and DMDHIQ + (200 μM) reduced in vivo free radical formation and reduced DA catabolism [13]. We also reported that (*R,S*)-SAL (50 and 100 μM) rescued neuroblastoma SH-SY5Y cells from death induced by 300 μM of H_2_O_2_ and by 50 μM of 6-hydroxydopamine (6-OHDA) [14].

Thus, the aims of the present study were three-fold: to compare the neuroprotective/neurotoxic potential of SAL with MPP^+^ in vitro, and in vivo, to examine morphological changes in the intestinal cross-sections as well as to search for basic metabolic alterations after single or continuous intraperitoneal SAL administration. This knowledge may provide a better understanding of the mechanisms related to peripheral dopaminergic dysregulation and conceivably improve animal modeling of PD.

## Methods

### In Vitro Experiments

#### Cell Culture and Reagents

SH-SY5Y (ATCC® CRL-2266TM) cell line was purchased from ATCC (American Type Culture Collection, USA). *(R,S)*-salsolinol (SAL) of purity ≥ 99% was obtained from Cayman Chemical (USA). MPP^+^ (1-methyl-4-phenylpyridinium iodide) of purity ≥ 98% was purchased from Angene (India). The CellTiter 96® AQueous Non-Radioactive Cell Proliferation Assay with a novel tetrazolium compound, namely 3-(4,5-dimethylthiazol-2-yl)-5-(3-carboxymethoxyphenyl)-2-(4-sulfophenyl)-2H-tetrazolium (MTS) was purchased from Promega (Madison, USA). Rhodamine 123 and Hoechst 33,258 were purchased fro Sigma-Aldrich (Merck LifeScience, Poland) and ThermoFisher Scientific (USA), respectively.

#### MTS Assay

SH-SY5Y CRL-2266™ cells were seeded in 96-well transparent plate at a concentration of 0.7 × 10^4^ cells/well in 100 μl culture medium and cultured for 48 h to reach 70% confluence. The cells were preincubated first for 1 h with SAL at the final concentration 50 μM and next MPP^+^ (1000 μM final concentration) was added. After 48 h of incubation, the MTS labelling mixture was added to each well and cells were further incubated under the same conditions for 5 h. The absorbance was measured using a microplate reader EnSpire (PerkinElmer, USA) at 490 nm. Measurements, obtained from two independent experiments, were performed in quadruplicate and were analyzed by one way ANOVA followed by Bonferroni’s comparison test (GraphPad Prism™ software (version 8.0, USA). Statistical significance was set at p < 0.0001 (***) in comparison with the positive control: 1000 μM MPP^+.^ Results are shown as mean ± standard deviation (SD).

#### Fluorescent Microscopy

SH-SY5Y cells were seeded and incubated with SAL and MPP^+^ according to the same procedure as described above. After 48 h of incubation the cells were rinsed with PBS and the mixture containing 10 µM of rhodamine 123 and 10 µM of Hoechst 33,258 was added and incubated at 37 °C, 5%CO_2_ for 30 min. Representative pictures were taken next by a fluorescence microscope Leica DMi8 (20x). For calculation of mitochondrial membrane potential (MMP) the fluorescence intensity of cells stained by rhodamine 123 was measured by fluorescence microscope Leica DMi8. The average fluorescence was measured from at least 10 selected cells, 3 different wells and 2 independent experiments. Statistical significance was set at p < 0.001 (∗ ∗ ∗) in comparison with the positive control: MPP^+^ (1000 μM).

### In Vivo Experiments

#### Animal Modeling

Male Wistar rats (Jagiellonian University Medical College Animal Laboratory, Krakow, Poland), with the initial body weight 214 g–318 g, were individually housed, in the same optimal conditions (temperature maintained at 23 ± 2 °C, and on a 12:12 h dark/light cycle), with solid chow (standard diet, 2.86 kcal/g, Labofeed, Poland) and tap water provided ad libitum. The Jagiellonian University Bioethical Committee approved the experiment (ethical approval number—67/2009). The study was carried out following ethical, regulatory, and scientific principles, and reported according to ARRIVE guidelines [15]. All possible means were taken to minimize animal suffering.

**Fig. 1:**
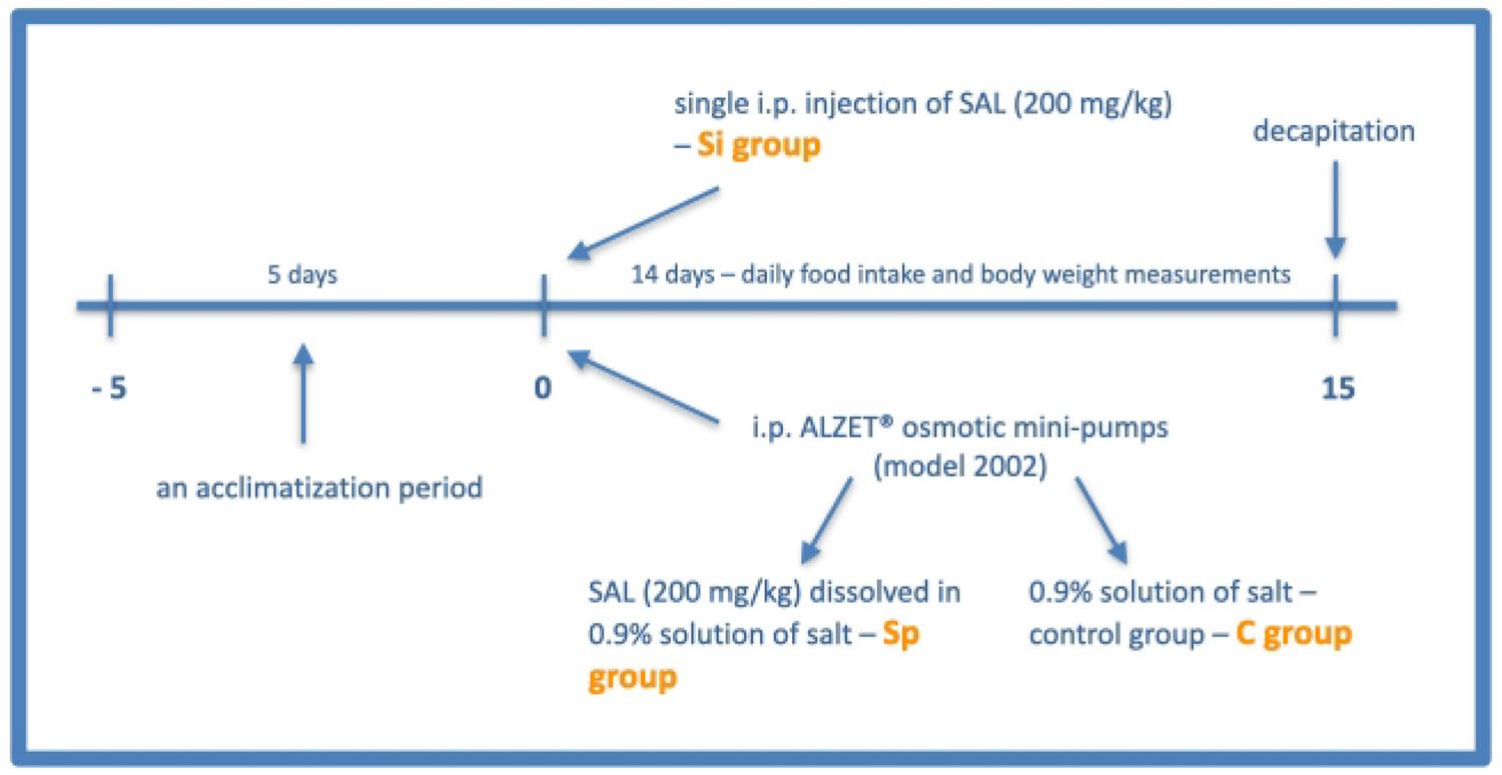
The general timeline of the in vivo experiment

Rats (n = 22) were randomly subjected to SAL administration or served as control (0.9% solution of salt administered via ALZET® osmotic mini-pumps). *(RS)*-SAL (salsolinol hydrochloride, Cayman Chemicals, USA) in the total dose of 200 mg/kg was dissolved in 0.9% solution of salt and was delivered intraperitoneally (i.p.) by either continuous ALZET® osmotic mini-pumps (ALZET, Durect, USA) – Sp group (n = 8) for two weeks or by a single injection—Si group (n = 8) at the begining of the experiment. Intraperitoneal mini-pumps’ implantation was performed under general anesthesia induced with sodium pentobarbital given i.p. at a dose of 0.25 mg/kg (Vetbutal, Biowet, Poland). During the study, daily food intake (g) and body weight (g) were measured each morning (Fig. 1). The general health status and motor function of the experimental animals were also evaluated daily during handling and by observing in-cage behavior.

At the end of the experiment, i.e., in the morning after 14 days, animals were euthanized by decapitation. Jejunal tissue fragments were collected for the morphological analysis and blood samples for the biochemical analysis. Immediately after euthanasia epididymal fat pads, located between the cauda epididymis and the distal extremity of the testis, were also dissected from each rat and weighed. The epididymal fat pad (EFP) over final body weight ratio (‰) was calculated by dividing the fat pad weight by the final body weight. Feed efficiency ratio (%) was calculated according to the following equation: [body weight gain (g)/total food intake (g)] × 100.

#### Biochemical Analysis

Blood samples from the jugular vessels were collected immediately after decapitation into plastic tubes and incubated for 30 min at 4 °C for clot formation. After centrifugation at 1500 g for 20 min at 4 °C (Megafuge 1.0R, Heraeus Instruments), serum samples were collected and frozen at − 80 °C until further analysis. Serum aliquots were prepared from each sample to measure glucose, aspartate (AST) and alanine (ALT) aminotransferases, triglycerides (TG) as well as low (LDL) and high-density (HDL) lipoprotein levels. All measurements (Chemistry Immuno Analyzer Olympus AU 600) were performed in duplicate.

#### Morphological Analysis

Fresh tissue specimens were rinsed thoroughly with PBS (phosphate-buffered saline, 0.01 M, pH  7.4), fixed with 9% phosphate-buffered paraformaldehyde, routinely processed, and embedded in paraffin (formalin-fixed paraffin-embedded, FFPE). Solidified paraffin blocks were cut into 5 μm thick sections and placed on slides with increased adhesion (Super Frost plus, Thermo Scientific, USA).

##### Gross Histology and Histochemical Detection of Collagen Deposition

Intestinal full-thickness FFPE sections were deparaffinized, rehydrated and processed for the routine hematoxylin and eosin (H&E) staining to evaluate the gross tissue organization as well as Masson’s Trichrome staining to demonstrate collagen (blue) and muscle fibers (red). All needed reagents were purchased from Sigma-Aldrich (Merck LifeSciences, Poland).

##### Immunohistochemistry and Immunofluorescence

Firstly, intestinal full-thickness FFPE sections were deparaffinized and rehydrated. Secondly, to obtain satisfactory immunostaining results, tissue antigenicity was retrieved by heat-induced epitope retrieval method (citrate buffer solution, pH 6.0 for 20 min at 96 °C). Further, sections were preincubated with hydrogen peroxide and/or appropriate non-immune normal sera with 0.5% Triton X-100 (room temperature for 15 min), and primary antibodies diluted in phosphate-buffered saline (PBS) overnight at 4 °C. The following day, sections were exposed to appropriate biotinylated immunoglobulins, peroxidase-labelled streptavidin complex and 3.3′-diaminobenzidine tetrahydrochloride or fluorophore-conjugated secondary antibodies diluted in PBS, at room temperature for 1 h. All reagents, including primary and secondary antibodies, used for immunoenzymatic and immunofluorescent assessment are given in Table 1.

**Table 1:** Regents used for immunoenzymatic and immunofluorescent assessment

Primary antibodies	Dilution	Reference	Vendor
Anti-PGP 9.5 (rabbit polyclonal)	1:100	Z5116	Dako, Denmark
Anti-S100 (rabbit polyclonal)	ready to use	760–2523	Ventana Medical Systems, USA
Anti-TH (rabbit polyclonal)	1:150	AB438RA01	Clone Cloud, USA
Secondary antibodies			
Alexa Flour-488 (goat anti-rabbit)	1:800	111–545-144	Jackson Immunoresearch, USA
Reagents			
Citrate buffer IHC Select pH 6.0	1:10	21,545	Merck, Germany
Phosphate buffer (10x)	1:10	42,595.01	Serva, Germany
Triton X-100	0.1%	X100	Sigma-Aldrich, Merck Life Science, Poland
Protein Block Serum Free	ready to use	X0909	Dako, Denmark
Antibody Diluent with background reducing components		S3022	
OptiView DAB IHC Detection Kit		760–700	Ventana Medical Systems, USA
Fluoroshield with DAPI, mounting medium		sc-24941	Santa Cruz, USA
Fluoroshield, mounting medium		sc-516212	
Neutral buffered formaldehyde, ethanol, xylene		-	ChemPur, Poland
DPX Mountant		6522	Sigma-Aldrich, Merck Life Science, Poland

#### Image Analysis and Statistical Analysis

All sections were analyzed qualitatively (H&E, S100-immunostaining, TH-immunostaining) and/or quantitatively (Masson’s Trichrome, PGP 9.5-positive enteric ganglia) by two independent investigators (M.K.Ł and V.A., blinded to avoid bias) using Axiophot (Zeiss, Germany) light equipped with ProgRes C12 plus digital camcorder (Jenoptik, Germany) and Multiscan 18.03 software (CSS, Poland) or MN800FL epifluorescence microscope (OptaTech, Poland) equipped with the Olympus 178 DP74 camera and CellSens software (Olympus Corporation). At least 20 consecutive fields of vision from two non-consecutive cross-sections from each animal were used for the assessment of the staining of the intestinal wall. Data obtained from all the examined fields for each rat were averaged and reported as mean values ± SD for each experimental group. Results were either described qualitatively or assessed quantitatively (or semi-quantitatively) using a one-way analysis of variance (ANOVA) followed by a post hoc Tuckey’s test. Statistical tests were performed using STATISTICA 13.3 software package (TIBCO Software Inc., USA; licensed to Jagiellonian University Medical College). Statistical significance was set at p < 0.05. All data are expressed as the mean and standard deviation (SD).

## Results

### In Vitro Results

**Fig. 2:**
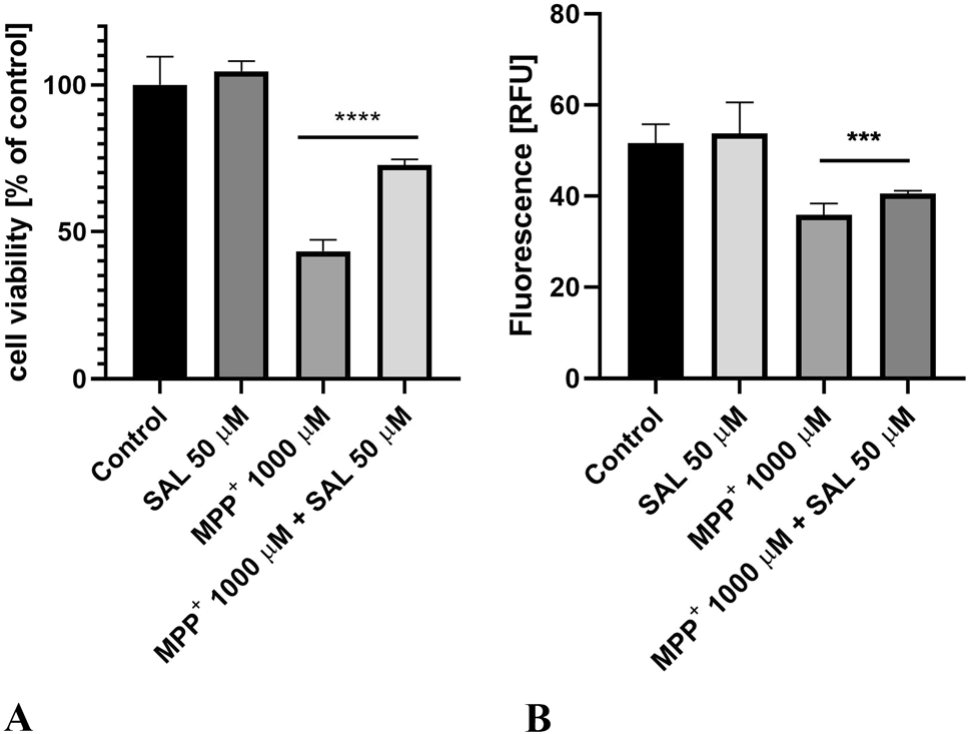
**A** Results of MTS test showing the neuroprotective effect of SAL (50 µM) on SH-SY5Y neuroblastoma cells viability damaged by MPP^+^ (1000 μM) after 48 h of incubation. Statistical significance was set at p < 0.0001 (∗ ∗  ∗ ∗) in comparison with MPP^+^ (1000 μM). **B** Protective effects of SAL on loss of mitochondrial membrane potential (MMP) in SH-SY5Y neuroblastoma cells. The fluorescence intensity was measured by fluorescence microscope Leica DMi8. Statistical significance was set at p < 0.001 (∗ ∗ ∗) in comparison with MPP^+^(1000 μM)

MTS assay showed (Fig. 2A.) statistically significant (p < 0.001) increase in viability of SH-SY5Y neuroblastoma cells treated with MPP^+^ (1000 μM) and SAL (50 µM) in comparison with the cells treated with MPP^+^ (1000 μM) alone (positive control). What is more, mitochondrial membrane potential (MMP) was monitored using rhodamine 123, a cell permeable cationic fluorescent dye that preferentially partitions into mitochondria based on the highly negative MMP. The fluorescence intensity (Fig. 2B.) was significantly increased in SH-SY5Y neuroblastoma cells treated with MPP^+^ (1000 μM) and SAL (50 µM) in comparison with the cells treated with MPP^+^ (1000 μM) alone (positive control).

**Fig. 3:**
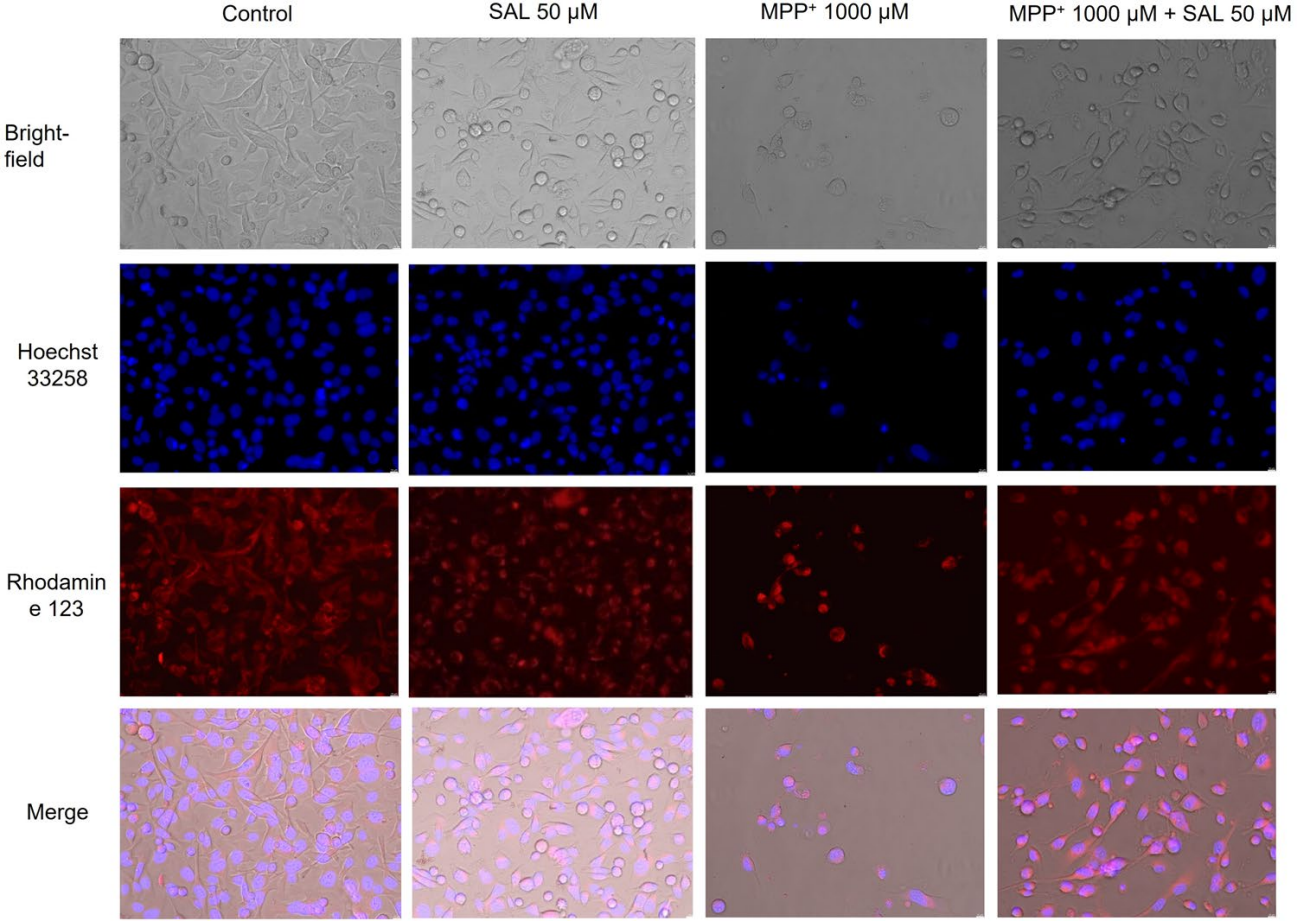
SH-SY5Y neuroblastoma cells exposed for 48 h to 50 μM SAL, 1000 μM MPP^+^ or 1000 μM MPP^+^ together with 50 μM SAL. Representative pictures were taken by a fluorescence microscope Leica DMi8 (original magnification 200x)

Photomicrographs of SH-SY5Y neuroblastoma cells treated with MPP^+^ alone or with MPP^+^ and SAL, and stained with Hoechst 22,258 (nuclear stain, emits fluorescence when bound to dsDNA) and rhodamine 123 (cationic fluorescent dye, easily sequestered by active mitochondria without cytotoxic effects) are presented in Fig. 3.

### In Vivo Results

#### Metabolic and Biochemical Analysis

None of the experimental rats died or showed any visible disturbances in gross motor function or discomfort to any degree. Feed efficiency ratio, which is a description of growth as a function of feed intake, was significantly lower in both SAL-treated groups in comparison to the control group, however, epididymal fat pad weight over final body weight was lower only in Si (single i.p. injection of SAL) group (p = 0.38). Glucose and triglycerides levels were significantly lower in Si group (p = 0.0034 and p = 0.02, respectively), while LDL to HDL ratio and aspartate to alanine aminotransferase (AST/ALT) ratio levels were significantly higher in Sp (continuous i.p. SAL delivery) group (p = 0.0003 and p = 0.035, respectively), in comparison with the control group. All the above-mentioned results are given in Table 2.

**Table 2:** Feed efficiency ratio, epididymal fat pad (EFP) weight over final body weight as well as glucose, triglicerydes (TG), LDL/HDL, and AST/ALT levels measured in serum

Group	Feed efficiency ratio (%)	EFP weight/final body weight ratio (‰)	Glucose (mmol/l)	TG (mmol/l)	LDL/HDL ratio	AST/ALT ratio
Si	17.9* ± 2.9 (p = 0.018)	**9.57 ± 1.47** (p = 0.38)	4.28* ± 0.24 (p = 0.0034)	0.17* ± 0.09 (p = 0.02)	0.2124 ± 0.054	3.67 ± 1.14
Sp	17.4* ± 1.8 (p = 0.006)	10.77 ± 0.52	7.48 ± 0.84	1.35 ± 0.45	1.394* ± 0.465 (p = 0.0003)	4.27* ± 1.33 (p = 0.035)
C	21.5 ± 2.4	10.96 ± 0.98	6.32 ± 1.45	2.45 ± 1.71	0.458 ± 0.431	2.93 ± 0.62

Experimental groups: *Si* SAL delivery via single i.p. injection, *Sp* SAL delivery via osmotic mini-pumps, *C* control group

*vs. control (C) animals

#### Gross Histology

**Fig. 4:**
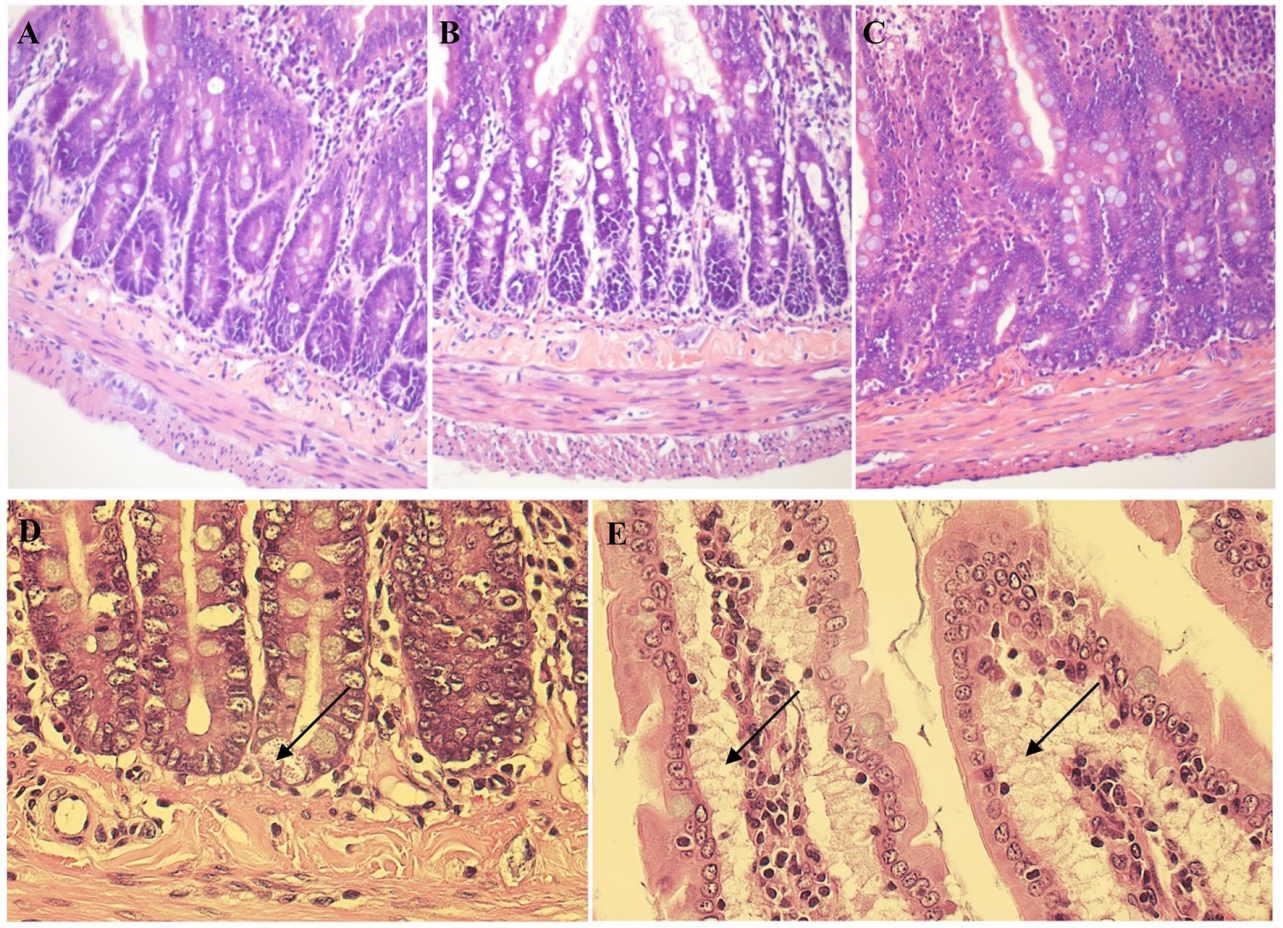
Gross morphology of jejunal cross-sections. Representative pictures of H&E-stained samples of full-thickness, cross-sectioned jejunum obtained from control (**A**) and SAL-treated (**B**-**E**) rats, showing the GI architecture across all experimental groups (upper panel) together with enhanced epithelial secretion (**D**) at the crypt base and subepithelial spaces (**E**) observed in the Sp group (marked with arrows). Original magnification 200x (objective: 20x, NA 0.45, upper panel) or 600x (objective: 60x, NA 0.85, lower panel)

**Fig. 5:**
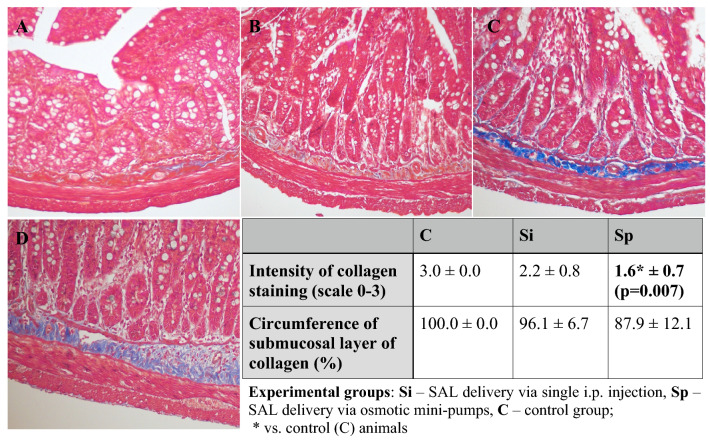
Collagen content in the jejunal cross-sections. Representative pictures of Masson’s Trichrome-stained samples of full-thickness, cross-sectioned jejunum obtained from SAL-treated (**A**-**C**) or control animals (**D**), showing collagen (blue) depletion (A–Sp group, B–Si group) or increased collagen deposition observed in the tunica mucosa/submucosa and tunica muscularis (C–Si group). Original magnification 200x (objective: 20x, NA 0.45). Table displays the assessment of collagen content expressed as the intensity of submucosal collagen staining and the circumference of submucosal layer of collagen

Gross morphology of the jejunal cross-sections did not show any significant inflammatory tissue alterations in SAL-treated rats in comparison to control animals (Fig. 4.), apart from: subepithelial spaces and enhanced epithelial secretion at the crypt base, both observed in the Sp (continuous i.p. SAL delivery) group. What is more, Masso’s Trichrome stain of the jejunal cross-sections revealed considerably depleted collagen content throughout the GI wall in the Sp group of SAL-treated rats (Fig. 5.). Sections from Si (single i.p. injection of SAL) group, though, were inconsistent and variable, including either collagen depletion similar to Sp group or increased collagen deposition observed in the tunica mucosa/submucosa and tunica muscularis.

#### Immunofluorescence and Immunohistochemistry

**Fig. 6:**
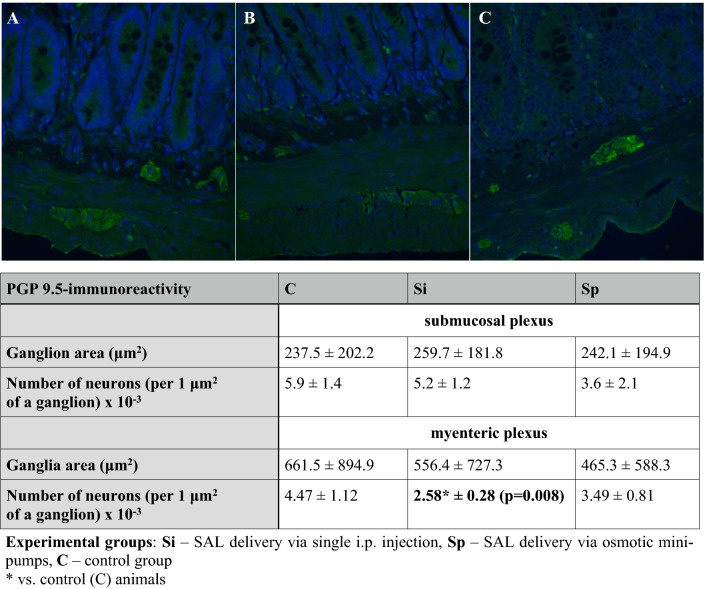
PGP 9.5-imunoreactivity in the jejunal cross-sections. Representative pictures of PGP 9.5-stained and DAPI-counterstained samples of full-thickness, cross-sectioned jejunum obtained from control (**A**) or SAL-treated animals (**B**–Si group, **C**–Sp group of rats). Original magnification 400x (objective 40x, NA 0.75). Table displays the assessment of PGP 9.5 immunoreactivity in the enteric nervous system, expressed as ganglionic area and neurons’s density in the ganglionic area

Ganglionic area measured as PGP 9.5-immmunoreactivity (Fig. 6.) was the lowest in the myenteric plexus in the Sp (continuous i.p. SAL delivery) group and remained unaffected in the submucosal plexus. SAL-treatment also significantly diminished the number of neurons density in the enteric ganglia, especially in the myenteric plexus from the Si (single i.p. injection of SAL) group in comparison to control animals.

**Fig. 7:**
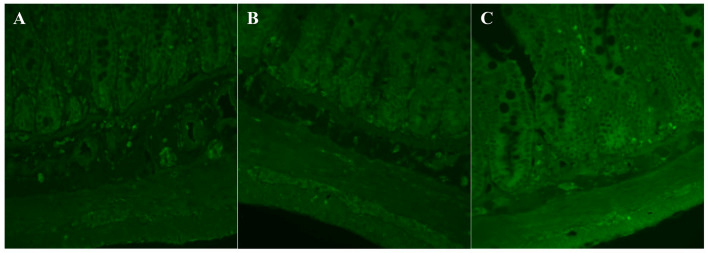
TH-imunoreactivity in the jejunal cross-sections. Representative pictures of TH-stained samples of full-thickness, cross-sectioned jejunum obtained from control (**A**) or SAL-treated animals (**B**–Si group, **C**–Sp group of rats). Original magnification 400x (objective 40x, NA 0.75)

Tyrosine hydroxylase (TH)-immunoreactivity was detected in all layers of the GI wall (Fig. 7.), and was expressed in neuronal and non-neuronal cells. TH is the rate limiting enzyme in DA synthesis and is used as a marker of catecholaminergic neurons, including dopaminergic ones. An increased epithelial TH-immunoreactivity in the intestinal crypts (especially at the at the crypt base) was observed in cross-sections from both SAL-treated groups. What is more, cross-sections from the Sp group were characterized by an increased TH-immunoreactivity in the longitudinal muscle layer and an increased autofluorescence in both muscle layers. TH-immunoreactivity in the enteric ganglionic area was infrequent, more pronounced in the submucosal than in the myenteric plexus, yet comparable between the groups.

It should also be mentioned that unstained jejunal FFPE sections from SAL-treated groups of rats were characterized by high autofluorescence emission in blue and green spectra, increased by half in Si (single i.p. injection of SAL) group and two times in Sp (continuous i.p. SAL delivery) group in comparison to the control group.

**Fig. 8:**
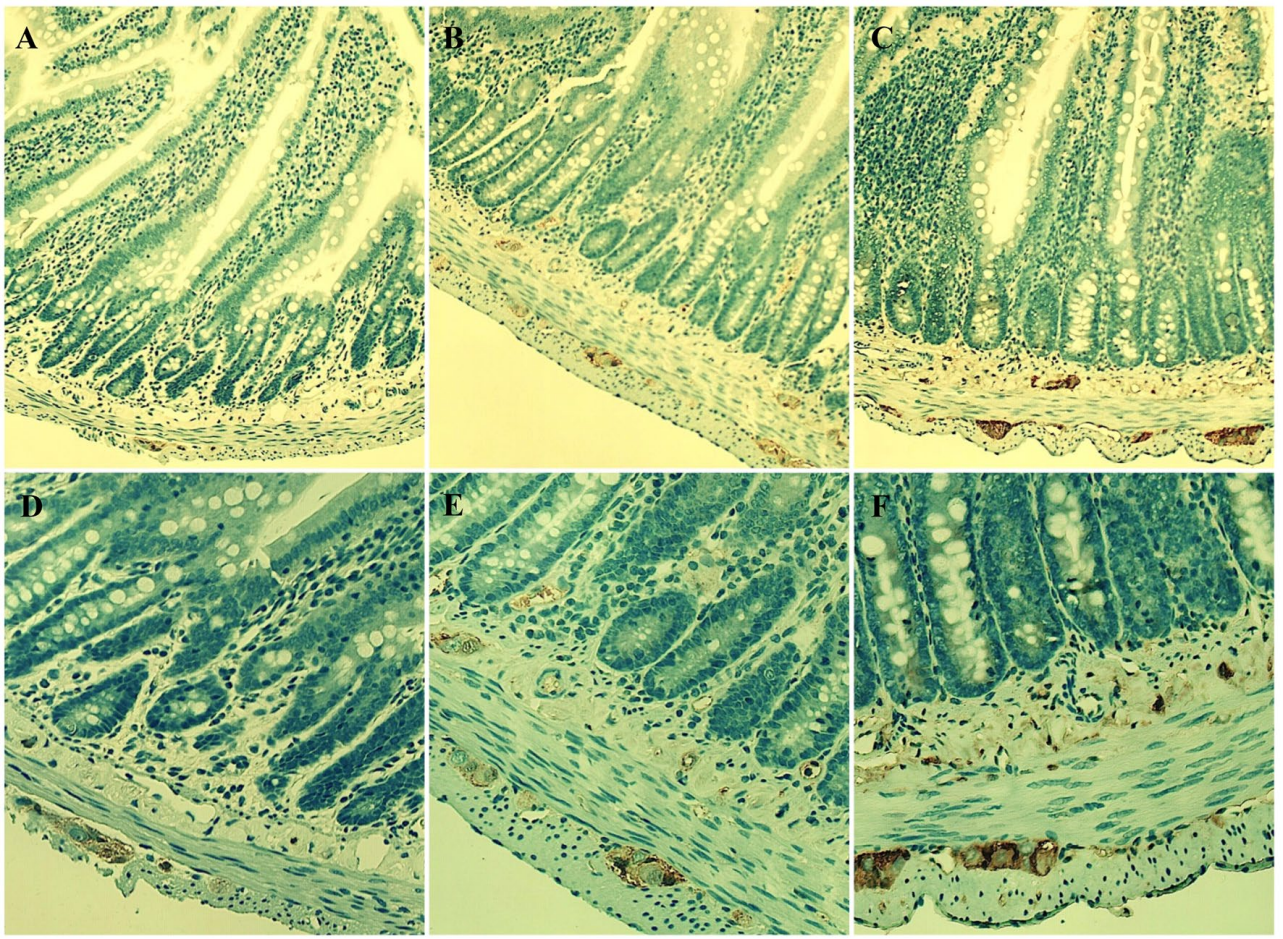
S100-imunoreactivity in the jejunal cross-sections. Representative pictures of S100-stained samples of full-thickness, cross-sectioned jejunum obtained from control (**A**, **D**) or SAL-treated animals (**B**, **E**–Si group, **C**, **F**–Sp group of rats). Original magnification 100x (upper panel, objective 10x, NA 0.30) or 200x (lower panel, objective 20x, NA 0.05)

S100 immunoreactivity was observed within the enteric ganglia in both plexuses**,** and SAL-treatment substantially increased its expression, especially in the Sp group, in comparison with the control animals, in both enteric plexuses in the same manner (Fig. 8). S100 is a calcium-binding protein and is commonly used as specific marker in order to identify enteric glial cells.

## Discussion

In the present study, SAL (50 μM) significantly rescued neuroblastoma cells from cell death mediated by MPP^+^ (1000 μM), which supports the neuroprotective potential of SAL. Previously, we also reported that SAL in the concentration range of 10–250 μM did not show any significant release of lactate dehydrogenase from necrotic SH-SY5Y cells and was able in the concentration range of 50–100 μM to rescue SH-SY5Y cells from death induced by H_2_O_2_ and 6-OHDA. SAL was found to significantly reduce reactive oxygen species level in SH-SY5Y cells treated by 500 μM H_2_O_2_ and the caspase activity induced by 300 μM of H_2_O_2_ [10]. On the other hand, alike the majority of published data [for review see 7], Arshad et al. [16] reported that MPP^+^, SAL, and 2[N]-methyl-(*R*)-SAL (NMSAL)-mediated cell death followed apoptotic pathways, and the lethal dose values determined for MPP^+^, SAL, and NMSAL were 500, 1500, and 1000 mM, respectively [16]. Yet, from the chemical point of view, SAL could indeed exhibit neuroprotective properties due to the presence of catechol (1,2-dihydroxybenzene) moiety. The relatively high antioxidant activity of catechol can be explained by the high electron-donating effect of one hydroxyl group to the other [17, 18]. And it was Maruyama et al. [13] who for the first time reported that (*R*)-SAL and the isoquinolinium (DMDHIQ^+)^ ion reduced hydroxyl radical production from dopamine autoxidation in vitro [13]. SAL is a tetrahydroisoquinoline derivative with an asymmetric center at C-1. In the human brain, SAL is converted by N-methyltransferase to produce 1,2-dimethyl-6,7-dihydroxy-1,2,3,4-tetrahydroisoquinoline (2[N]-methyl-(*R*)-SAL), which is oxidized to produce 1,2-dimethyl-3,4-dihydroisoquinoline, and further metabolized by MAO-A/B (or amine oxidases sensitive to semicarbazide [7]) to produce the charged quaternary form such as DMDHIQ^+^ [19]. Thus, the neurobiological role of SAL could be dose-dependent and possibly stereoisomer-dependant, yet whether SAL could undergo similar conversion in the peripheral/enteric nervous system remains unknown. Based on our results we suggest that commercially available racemic SAL in the doses below 100 μM might serve as an excellent reference compound for in vitro studies related to neuroprotection/neurodegeneration [20].

In the present study, we also demonstrated that neuroprotective properties of racemic SAL observed in neuroblastoma SH-SY5Y cell line may not necessarily and straightforwardly translate in vivo. It is likely that local GI exposition to exogenous SAL exceeded the dose applied in vitro. Thus, the dose might indeed determine the neurobiological properties of SAL both centrally [21, 22] and peripherally. Foremostly, our in vivo results showed that SAL given intraperitoneally could alter GI homeostasis, yet without any obvious signs of inflammation. According to the morphological assessment, the number of PGP 9.5–positive neurons and the ganglionic area in the myenteric plexus were decreased in SAL-treated animals in comparison with the control animals. However, in the submucosal plexus only the neuronal density was diminished in SAL-treated rats, yet insignificantly. So far, studies on age-associated neurodegeneration throughout the GI tract have given variable results, which are further prone to inter-study variation due to the use of different animal models, strains of animals, and analyzed regions of the gut [23]. Interestingly, the density of enteric innervation can determine the severity of intestinal inflammation [24]. Previously, we reported a decrease in the myenteric neuron count, the mean size of the neuron body, the area of ganglia and the diameter of nerve strands in LMMP preparations from SAL-treated rats. The number of myenteric nitrergic neurons was lower in the SAL-treated groups, while the number of cholinergic neurons remained unchanged [14]. What is more, image analysis of duodenal cross-sections revealed decreased c-Kit expression in the SAL-injected rats [11]. So far, aging of the enteric nervous system (ENS) was characterized by the loss of cholinergic enteric neurons and interstitial cells of Cajal, whereas inhibitory neurons appeared to be unaffected [25, 26]. Such imbalanced proportion of neuronal subtypes should result in dysregulated bowel motility [27]. In PD, the ENS is vulnerable to neurodegeneration but it remains unclear if the disease targets any specific sites or neuronal subtypes [28, 29]. The loss of dopaminergic neurons in the substantia nigra pars compacta (SNpc), being the pathological hallmark of PD, is not necessarily accompanied by the neurodegeneration of enteric dopaminergic neurons. While SNpc dopaminergic neurons are protected by the blood–brain barrier, enteric dopaminergic neurons lack a blood barrier and are continuously exposed to potentially toxic elements that may translocate across the epithelium from the gut lumen and are in close proximity to the gut microbiome [29]. Previously, we reported no TH-immunopositive cell bodies together with a dense architecture of TH-immunopositive nerve fibers in LMMP preparations, and no differences were observed either in control or SAL-treated rats [14]. Here, we report limited TH-immunoreactivity in the enteric ganglionic area of both plexuses, still comparable between all experimental groups. According to in vitro, in vivo, and ex vivo studies, SAL has the ability to affect dopaminergic neurotransmission via inhibition of tyrosine hydroxylase [30–32]. SAL could also bind to peripheral dopamine receptors (unpublished, theoretical data based on computer modeling) and thus possibly in the GI tract modulate exocrine secretion, fluid absorption, motility, blood flow, cytoprotection, and GI immune regulation [8], yet with unknown affinity or type of interaction. What is more, the affinity of SAL to GI dopamine receptors, alike DA [33], could be dose-dependent. Our gross morphological evaluation did not reveal any clear signs of jejunal inflammation in SAL-treated rats regardless of the mode of administration, except for intensified autofluorescence. What we did observe was enhanced epithelial secretion at the crypt base where Paneth cells reside. Those monoamine-secreting epithelial cells, apart from their canonical antimicrobial function, have also been shown to play an essential niche role in supporting GI stem cells [34]. Since Paneth cells express TH [35], SAL could alter cryptal homeostasis. Foremostly, administration of SAL was associated with an increased ganglionic expression of a calcium-binding glial protein S100, especially after continuous SAL administration. Enteric glial cells, based on morphology and protein expression, such as glial fibrillary acidic protein, S100, or proteolipid-protein-1, can be differentiated into several distinct glial types [36]. However, it remains uncertain, if an increase in the population of enteric glial cells, which are active participants in neuroimmune regulation and intestinal barrier maintenance [24], exerts any anti-inflammatory action. The reactive gliosis is likely responsible for PD-related neuroinflammation and all associated pathological changes in the ENS [37]. The up-regulation of S100-positive sub-epithelial colonic glial cells was also observed in nigrostriatally 6-OHDA-injected rats, and most probably resulted from the impairment of tight-junctions and the proliferative trend of colonic epithelium. However, that pathological remodeling of the colon was further associated with submucosal inflammation together with enhanced fibrotic deposition [38]. Conversely, we predominantly observed depleted submucosal collagen content following SAL-administration. Thus, SAL might have either influenced fibroblasts or have bound to collagen fibers chemically interfering with the Masson’s trichrome stain. Collectively, GI consequences of peripheral SAL-administration advocate the need to investigate this dopaminergic compound as an enteric microglial modulator. This need is further stressed by the fact that enteric glial cells regulate intestinal epithelial barrier integrity and are able to establish bidirectional interactions with the GI microbiota, and thus their role in the pathogenesis and progression of PD have been suggested. Clinical data clearly support such hypotheses [37].What is more, increased levels of enteric glial-related pro-inflammatory markers observed in the colon of PD patients, during the earliest stages of the disease, decisively imply that the initial glial response is characteristic for the early stage disease [39]. Then, such early reactive enteric gliosis might promote local neuroinflammation that could ascend to the central nervous system through glial hemichannels, and possibly via vagal nerve fibers, which again supports the fundamental role of the gut-brain axis in the pathogenesis of PD [37].

Thirdly, all SAL-treated animals also presented decreased feed efficiency ratio, which could be a direct consequence of imbalanced GI dopaminergic homeostasis and impaired nutrients absorption. However, EFP weight over final body weight was only significantly decreased in Si (single i.p.injection of SAL) group of animals. Other metabolic consequences of SAL-administration included diminished glucose and TG serum levels in Si group and increased LDL/HDL ratio in Sp (continuous i.p. SAL delivery) group of animals. Peripheral DA stimulated glucose uptake with its receptors being differentially involved in glucose uptake in insulin-sensitive tissues [40], and was also produced by rat adipocytes isolated from mesenteric adipose tissue [41]. Thus, if DA is able to modulate insulin signaling, glucose uptake, and activation of metabolic pathways in the periphery [42], SAL via DA receptors could interfere with those pathways and impact on glucose and lipid metabolism. These modulatory effects of SAL clearly depend on a dose or treatment duration or a mode of administration, and require confirmation by further focused studies. At the same time, neurodegenerative diseases, including PD, are also characterized by a range of metabolic alterations, such as type 2 diabetes mellitus, obesity, and non-alcoholic fatty liver disease [43]. Thus, the use of SAL in modeling enteric neurodegeneration is indeed worthy of more consideration.

## Conclusion

According to our study, low doses of SAL exhibited neuroprotective properties against MPP^+^ in SH-SY5Y cell line, which further supports the use of SAL as a reference compound for i*n vitro* studies. In vivo results give insight into our understanding of gastrointestinal remodeling following intraperitoneal SAL administration, pointing towards the possible use of this DA derivative to model a microglial-related enteric neurodegeneration and dopaminergic dysregulation.

## Data Availability

Data are available from authors upon reasonable request.

## Abbreviations

ALT: Alanine aminotransferase
ASP: Aspartate aminotransferase
DA: Dopamine
DMDHIQ^+^: 1,2-Dimethyl-6,7-dihydroxyisoquinolinium ion
ENS: Enteric nervous system
EFP: Epididymal fat pad
FFPE: Formalin-fixed paraffin-embedded
GI: Gastrointestinal
HDL: High-density lipoprotein
i.p.: Intraperitoneally
LDL: Low-density lipoprotein
L-DOPA: Levodopa, L-3,4-dihydroxyphenylalanine
LMMP: Longitudinal muscle myenteric plexus
MMP: Mitochondrial membrane potential
MPP^+^: 1-Methyl-4-phenylpyridinium ion
MPTP: 1-Methyl-4-phenyl-1,2,3,6-tetrahydropyridine
MTS: 3-(4,5-Dimethylthiazol-2-yl)-5-(3-carboxymethoxyphenyl)-2-(4-sulfophenyl)-2H-tetrazolium;
PD: Parkinson’s disease
PGP 9.5: Protein gene product 9.5–pan-neuronal marker
SAL: Salsolinol, 1-methyl-6,7-dihydroxy-1,2,3,4-tetrahydroisoquinoline
S100: A calcium-binding protein
TH: Tyrosine hydroxylase
6-OHDA: 6-Hydroxydopamine

## References

[1] Sandler M, Carter SB, Hunter KR, Stern GM (1973). Tetrahydroisoquinoline alkaloids: In vivo metabolites of L-dopa in man. Nature.

[2] Naoi M, Matsuura S, Takahashi T, Nagatsu T (1989). A N-methyltransferase in human brain catalyses N-methylation of 1,2,3,4-tetrahydroisoquinoline into N-methyl-1,2,3,4-tetra- hydroisoquinoline, a precursor of a dopaminergic neurotoxin, N-methylisoquinolinium ion. Biochem Biophys Res Commun.

[3] Naoi M, Maruyama W, Zhang JH, Takahashi T, Deng Y, Dostert P (1995). Enzymatic oxidation of the dopaminergic neurotoxin, 1(R), 2(N)-dimethyl-6,7-dihydroxy-1,2,3,4-tetra- hydroisoquinoline, into 1,2(N)-dimethyl-6,7-dihydroxyisoquinolinium ion. Life Sci.

[4] Naoi M, Maruyama W, Dostert P, Kohda K, Kaiya T (1996). A novel enzyme enantio- selectively synthesizes (R)salsolinol, a precursor of a dopaminergic neurotoxin, N-methyl(R) salsolinol. Neurosci Lett.

[5] Naoi M, Maruyama W, Akao Y, Yi H (2002). Dopamine-derived endogenous N-methyl-(R)- salsolinol: Its role in Parkinson’s disease. Neurotoxicol Teratol.

[6] Maruyama W, Sobue G, Matsubara K, Hashizume Y, Dostert P, Naoi M (1997). A dopaminergic neurotoxin, 1(R), 2(N)-dimethyl-6,7-dihydroxy-1,2,3,4-tetrahydroisoquinoline, N-methyl(R)salsolinol, and its oxidation product, 1,2(N)-dimethyl-6,7-dihydroxyisoquinolinium ion, accumulate in the nigro-striatal system of the human brain. Neurosci Lett.

[7] Kurnik-Łucka M, Panula P, Bugajski A, Gil K (2018). Salsolinol: an unintelligible and double-faced molecule-lessons learned from in vivo and in vitro experiments. Neurotox Res.

[8] Kurnik-Łucka M, Gil K, Kostrzewa RM (2021). Enteric Neurotoxicity and Salsolinol. Handbook of Neurotoxicity.

[9] Braak H, Rüb U, Gai WP, Del Tredici K (2003). Idiopathic Parkinson’s disease: possible routes by which vulnerable neuronal types may be subject to neuroinvasion by an unknown pathogen. J Neural Trans.

[10] Kurnik-Łucka M, Latacz G, Martyniak A, Bugajski A, Kieć-Kononowicz K, Gil K (2020). Salsolinol-neurotoxic or neuroprotective?. Neurotox Res.

[11] Banach T, Zurowski D, Gil K, Krygowska-Wajs A, Marszałek A, Thor PJ (2006). Peripheral mechanisms of intestinal dysmotility in rats with salsolinol induced experimental Parkinson’s disease. J Physiol Pharmacol.

[12] Kurnik M, Gil K, Bujak-Gizycka B, Madej J, Kaszuba-Zwoinska J, Bialas M, Bugajski A, Thor P (2013). Elevated interleukin-1β serum level after chronic peripheral salsolinol administration. Folia Med Cracov.

[13] Maruyama W, Dostert P, Naoi M (1995). Dopamine-derived 1-methyl-6,7- dihydroxyisoquinolines as hydroxyl radical promoters and scavengers in the rat brain: In vivo and in vitro studies. J Neurochem.

[14] Kurnik M, Gil K, Gajda M, Thor P, Bugajski A (2015). Neuropathic alterations of the myenteric plexus neurons following subacute intraperitoneal administration of salsolinol. Folia Histochem Cytobiol.

[15] Percie du Sert N, Ahluwalia A, Alam S, Avey MT, Baker M, Browne WJ, Clark A, Cuthill IC, Dirnagl U, Emerson M, Garner P, Holgate ST, Howells DW, Hurst V, Karp NA, Lazic SE, Lidster K, MacCallum CJ, Macleod M, Pearl EJ, Petersen OH, Rawle F, Reynolds P, Rooney K, Sena ES, Silberberg SD, Steckler T, Würbel H (2020). Reporting animal research: explanation and elaboration for the ARRIVE guidelines 2.0. PLoS Biol.

[16] Arshad A, Chen X, Cong Z, Qing H, Deng Y (2014). TRPC1 protects dopaminergic SH-SY5Y cells from MPP+, salsolinol, and N-methyl-(R)-salsolinol-induced cytotoxicity. Acta Biochim Biophys Sin (Shanghai).

[17] Heijnen CG, Haenen GR, Oostveen RM, Stalpers EM, Bast A (2002). Protection of flavonoids against lipid peroxidation: the structure activity relationship revisited. Free Radic Res.

[18] Heijnen CG, Haenen GR, van Acker FA, van der Vijgh WJ, Bast A (2011). Flavonoids as peroxynitrite scavengers: the role of the hy- droxyl groups. Toxicol in Vitro.

[19] McNaught KS, Carrupt PA, Altomare C, Cellamare S, Carotti A, Testa B, Jenner P, Marsden CD (1998). Isoquinoline derivatives as endogenous neurotoxins in the aetiology of Parkinson’s disease. Biochem Pharmacol.

[20] Łażewska D, Olejarz-Maciej A, Reiner D, Kaleta M, Latacz G, Zygmunt M, Doroz-Płonka A, Karcz T, Frank A, Stark H, Kieć-Kononowicz K (2020). Dual target ligands with 4-tert-butylphenoxy scaffold as histamine H3 receptor antagonists and monoamine oxidase B inhibitors. Int J Mol Sci.

[21] Panula P, Partanen S, Kaakkola S (1979). Fluorescence histochemical observations on the distribution of exogenous dihydroisoquinoline in the rat brain. Exp Brain Res.

[22] Możdżeń E, Kajta M, Wąsik A, Lenda T, Antkiewicz-Michaluk L (2015). Salsolinol, an endogenous compound triggers a two-phase opposing action in the central nervous system. Neurotox Res.

[23] Saffrey MJ (2014). Aging of the mammalian gastrointestinal tract: a complex organ system. Age.

[24] Margolis KG, Gershon MD (2016). Enteric Neuronal regulation of intestinal inflammation. Trends Neurosci.

[25] Phillips RJ, Powley TL (2007). Innervation of the gastrointestinal tract: patterns of aging. Auton Neurosci Basic Clin.

[26] Wang TH, Angeli TR, Ishida S, Du P, Gharibans A, Paskaranandavadivel N, Imai Y, Miyagawa T, Abell TL, Farrugia G, Cheng LK, O'Grady G (2021). The influence of interstitial cells of Cajal loss and aging on slow wave conduction velocity in the human stomach. Physiol Rep.

[27] Nezami BG, Srinivasan S (2010). Enteric nervous system in the small intestine: pathophysiology and clinical implications. Curr Gastroenterol Rep.

[28] Chalazonitis A, Rao M (2018). Enteric nervous system manifestations of neurodegenerative disease. Brain Res.

[29] Chalazonitis A, Rao M, Sulzer D (2022). Similarities and differences between nigral and enteric dopaminergic neurons unravel distinctive involvement in Parkinson's disease. NPJ Parkinsons Dis.

[30] Weiner CD, Collins MA (1978). Tetrahydroisoquinolines derived from catecholamines or DOPA: Effects on brain tyrosine hydroxylase activity. Biochem Pharmacol.

[31] Minami M, Takahashi T, Maruyama W, Takahashi A, Dostert P, Nagatsu T, Naoi M (1992). Inhibition of tyrosine hydroxylase by R and S enantiomers of salsolinol, 1-methyl- 6,7-dihydroxy-1,2,3,4-tetrahydroisoquinoline. J Neurochem.

[32] Briggs GD, Nagy GM, Dickson PW (2013). Mechanism of action of salsolinol on tyrosine hydroxylase. Neurochem Int.

[33] Feng YF, Lu Y (2021). Immunomodulatory effects of dopamine in inflammatory diseases. Front Immunol.

[34] Schmitt M, Schewe M, Sacchetti A, Feijtel D, van de Geer WS, Teeuwssen M, Sleddens HF, Joosten R, van Royen ME, van de Werken HJG, van Es J, Clevers H, Fodde R (2018). Paneth cells respond to inflammation and contribute to tissue regeneration by acquiring stem-like features through SCF/c-Kit signaling. Cell Rep.

[35] Penttilä A, Ahonen A (1969). Binding of 1–3,4-dihydroxyphenylalanine and dopamine in cytoplasmic granules of paneth cells. Experientia.

[36] Grundmann D, Loris E, Maas-Omlor S, Huang W, Scheller A, Kirchhoff F, Schäfer KH (2019). Enteric glia: S100, GFAP, and beyond. Anat Rec (Hoboken).

[37] Benvenuti L, D'Antongiovanni V, Pellegrini C, Antonioli L, Bernardini N, Blandizzi C, Fornai M (2020). Enteric glia at the crossroads between intestinal immune system and epithelial barrier: implications for parkinson disease. Int J Mol Sci.

[38] Pellegrini C, Ippolito C, Segnani C, Dolfi A, Errede M, Virgintino D, Fornai M, Antonioli L, Garelli F, Nericcio A, Colucci R, Cerri S, Blandini F, Blandizzi C, Bernardini N (2020). Pathological remodelling of colonic wall following dopaminergic nigrostriatal neurodegeneration. Neurobiol Dis.

[39] Clairembault T, Leclair-Visonneau L, Neunlist M, Derkinderen P (2015). Enteric glial cells: new players in Parkinson's disease?. Mov Disord.

[40] Ustione A, Piston DW, Harris PE (2013). Mini review: dopaminergic regulation of insulin secretion from the pancreatic islet. Mol Endocrinol.

[41] Vargovic P, Ukropec J, Laukova M, Cleary S, Manz B, Pacak K, Kvetnansky R (2011). Adipocytes as a new source of catecholamine production. FEBS Lett.

[42] Tavares G, Martins FO, Melo BF, Matafome P, Conde SV (2021). Peripheral dopamine directly acts on insulin-sensitive tissues to regulate insulin signaling and metabolic function. Front Pharmacol.

[43] Freude KK, Moreno-Gonzalez I, Rodriguez-Ortiz CJ, Baglietto-Vargas D (2022). Editorial: metabolic alterations in neurodegenerative disorders. Front Aging Neurosci.

