# Correction to: Salsolinol Protects SH-SY5Y Cells Against MPP^+^ Damage and Increases Enteric S100‐Immunoreactivity in Wistar Rats

**DOI:** 10.1007/s11064-023-03901-3

**Published:** 2023-02-28

**Authors:** Magdalena Kurnik-Łucka, Gniewomir Latacz, Joanna Goryl, Veronika Aleksandrovych, Krzysztof Gil

**Affiliations:** 1 Department of Pathophysiology, Faculty of Medicine, Jagiellonian University Medical College, Czysta 18, 31-121 Krakow, Poland; 2 Department of Technology and Biotechnology of Drugs, Faculty of Pharmacy, Jagiellonian University Medical College, Medyczna 9, Krakow, Poland

**Correction to: Neurochemical Research**

10.1007/s11064-022-03835-2

The original version of this article unfortunately contained the erroneous ethical approval number.

The correct ethical approval number is as follows: 103/2014. This has been corrected by publishing this correction article.

